# Combined Effect of Microplastics and Cd Alters the Enzymatic Activity of Soil and the Productivity of Strawberry Plants

**DOI:** 10.3390/plants11040536

**Published:** 2022-02-17

**Authors:** Andrés Pinto-Poblete, Jorge Retamal-Salgado, María Dolores López, Nelson Zapata, Angela Sierra-Almeida, Mauricio Schoebitz

**Affiliations:** 1 Faculty of Agronomy, Universidad de Concepción, Concepción 4070386, Chile; andrespinto@unach.cl (A.P.-P.); mlopezb@udec.cl (M.D.L.); nzapata@udec.cl (N.Z.); 2 Faculty of Engineering and Business, Universidad Adventista de Chile, Km 12 Camino a Tanilvoro, Chillán 3780000, Chile; 3 Instituto de Investigaciones Agropecuarias, INIA-Quilamapu, Avenida Vicente Méndez 515, Chillán 3800062, Chile; 4 Department of Botany, Faculty of Natural and Oceanographic Sciences, Universidad de Concepción, Barrio Universitario s/n, Concepción 4070386, Chile; angelasierra@udec.cl

**Keywords:** enzyme activity, chlorophyll fluorescence, polyethylene, contaminated soils, Andisol, root characteristics, *Fragaria × ananassa*

## Abstract

The synergistic effect between heavy metals and microplastics can affect soil properties as well as plant performance and yield. The objective of this study was to evaluate the combined effect of microplastics and cadmium on a soil–plant system. Specifically, we proposed to explore changes in soil microbiological activity, the growth and yield parameters of strawberry plants, and to evaluate the accumulation of these pollutants in the soil and root system. Plants were planted in clay pots under greenhouse conditions. The experiment was set up as a completely randomized design, with four treatments (Control; MPs; Cd; and Cd + MPs) and five replicates. The results showed that MPs and/or Cd affected plant growth, plant biomass, the number of fruits, root characteristics, dehydrogenase activity, acid phosphatase, and microbial biomass, and increased the accumulation of Cd in the roots and soil. The increased bioavailability of Cd, due to the presence of microplastics, could explain the observed negative effects on soil properties and the performance of strawberry plants.

## 1. Introduction

Microplastics (MPs) have been defined as particles smaller than 5 mm in size. These particles mainly originate from the disintegration of plastic mulch and household garbage [1]. The use of plastic has drastically increased in recent years [2], accounting for 10% of total waste generated around the world. While some plastic waste is recycled, most of it ends up in landfills or agricultural lands [3]. In fact, studies have estimated that up to 700,000 tons of MPs are deposited on agricultural lands in Europe and North America each year [4]. Concentrations as high as 7% of MPs have been reported in highly contaminated top soils [5], and the literature has described that MPs may have adverse effects on soil fauna, including earthworms, nematodes, collembolans, and mice [6].

Plastics, especially polyethylene (PE), are extensively used in agriculture, particularly in the use of plastic mulch films, row covers, greenhouse films, nursery pots, and silage bags [7]. Therefore, plastic mulches are important sources of MPs in soil [8]. Different studies have shown that plastic residues from mulch can release phthalate esters, inhibiting soil enzyme activity and altering microbial communities [9]. In strawberries (*Fragaria* x *ananassa* Duch), the use of plastic mulch is increasingly popular because of its ability to suppress weeds, reduce the use of chemical products for the control of pests and diseases [10], and improve plant growth conditions and water use efficiency [11]. It should be noted that the strawberry is one of the most produced and consumed berry species in the world. In 2020, the cultivated area in Chile was 1147 hectares with an annual production of 30,289 tons [12].

MPs can act as a vector for other contaminants such as human pathogens, pharmaceuticals, fungicides, and inorganic chemicals such as heavy metals [1]. The interactions between MPs and heavy metals might pose greater risks to soil organisms and soil quality [13]. Thus, the pollution caused by MPs was listed as one of the top environmental problems by the United Nations Environment Programme [14].

Soil contamination by heavy metals has been identified as a growing global problem in recent years, and its rapid increase in recent decades [15] has become an important threat to the environment and human health [16], resulting in the disappearance of vegetation and the alteration of soil microbial biodiversity [17], and also generating toxic effects on the physiology of plants [15]. Cadmium (Cd) is one of the most toxic heavy metals in agricultural soils. Its presence is caused by anthropogenic activities such as the use of fertilizers, agricultural sludge, or industrial activities [15]. When entering the trophic chain, Cd becomes toxic and carcinogenic to humans, and can accumulate in the liver and kidneys for more than 30 years [18]. The concentrations of Cd observed in the roots of the tomato, maize, and lettuce plants were 1.8, 0.15, and 2 mg kg^−1^, respectively [19,20,21]. It is important to note that according to the EU regulatory commission, the concentration of Cd in food should not exceed 1 mg kg^−1^ [22].

The synergistic effect between microplastics and heavy metals, such as Cd, can alter soil microbial communities [13,23], inhibit soil respiration, and affect enzyme activity [24], which plays a key role in the biodegradation of organic compounds in the soil [25]. There is evidence that microplastics and Cd affect plant development, generating significant changes in plant biomass by damaging the elemental composition of tissues and root traits [13]. However, to the authors’ best knowledge, there is no information on the effects resulting from the interaction between microplastics and Cd on the biological properties of soil, and on plant growth and the yield of strawberry plants. As stated above, the objective of this study was to evaluate the effect of microplastics and cadmium on changes in soil enzymatic activity and the productive parameters of strawberries.

## 2. Results

### 2.1. Plant Growth

**Figure 1:**
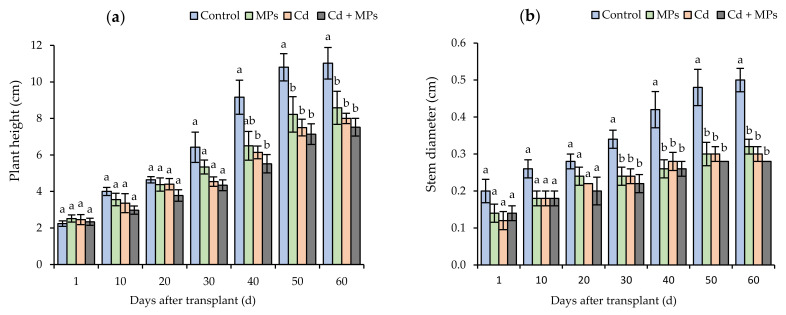
The effect of microplastics and/or cadmium on strawberries during plant growth for: (**a**) plant height (cm), (**b**) stem diameter (cm). Different lowercase letters indicate significant differences according to the LSD Fisher test (*p* < 0.05). Control: no contaminants added; MPs: microplastics added; Cd: cadmium added; Cd + MPs: cadmium and microplastics added.

Plant height and stem diameter were significantly affected by the separate applications of MPs and Cd, as well as their combined use. MPs significantly decreased plant height by 33.4% 40 days after strawberry plants were transplanted (Figure 1a). Regarding stem diameter, the control treatment was statistically different from day 30 (Figure 1b), and significant differences (*p* < 0.05) were observed between the control and the treatments containing contaminants (MPs, Cd, and Cd + MPs). Forty days after transplant, MPs, Cd, and Cd + MPs applications significantly decreased stem diameter compared to the control treatment, with reduction of 38%, 33% and 38%, respectively.

### 2.2. Photochemical Efficiency

**Figure 2:**
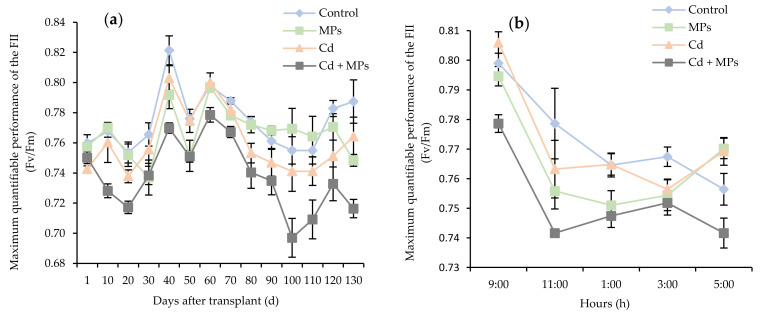
The maximum quantum yield of PSII II (Fv/Fm) measured in strawberry plants exposed to single and combined pollutants (**a**) during the entire experimental period, and (**b**) on an average day. Treatments correspond to: control (blue): no contaminants added; MPs (green): microplastics added; Cd (orange): cadmium added; and Cd + MPs (grey): cadmium and microplastics added.

The maximum photochemical efficiency of photosystem II at time intervals after transplant (Fv/Fm; Figure 2a) varied depending on the treatment. The control and MPs treatments recorded the highest FII quantum yield values, ranging from 0.74 to 0.82 and from 0.74 to 0.8, respectively. The Cd treatment performed similarly until day 70, when there was a slight decrease in the quantum yield of FII, while values from day 110 were close to the highest observed values. The Cd + MPs treatment presented lower values between 1–30 and 80–130 days after transplant compared to the other treatments, reaching the lowest value of 0.69 on day 100, while the lowest value did not exceed 0.78. In terms of maximum photochemical efficiency of photosystem II at different time points, the control, MPs, and Cd treatments recorded values above 0.79 at 9:00 am, while a lower value of 0.77 was obtained in the Cd + MPs treatment. As hours passed, the photochemical efficiency of photosystem II decreased until 1:00 pm, with values lower than 0.77 for all of the treatments. Similar to the previous parameter, the Cd + MPs treatment recorded the lowest value of 0.74 at 11 am From 1:00 to 5:00 pm, a more stable trend was observed, with levels ranging from 0.75 to 0.76 for all the treatments, except for the Cd + MPs treatment.

### 2.3. Morphological and Production Parameters

Regarding the parameters related to the production and reproduction of strawberries (Table 1), significant differences were observed in the total number of fruits. MPs and Cd + MPs had a lower yield, with a decrease of 54.55% (MPs, Cd + MPs) with respect to the control. Even though no significant differences were observed in parameters such as fruit weight, stolon length, the total number of stolons, and the number of inflorescences, the values recoded by the control treatment were higher for all the parameters studied. In terms of plant biomass, there were significant differences (*p* < 0.05) between the control and the treatments with contaminants added, with average values of 7.3, 4.2, 4.4, and 4.8 g plant^−1^ dry weight (dw) for the control, Cd, MPs, and Cd + MPs, respectively.

### 2.4. Root Characterization

**Figure 3:**
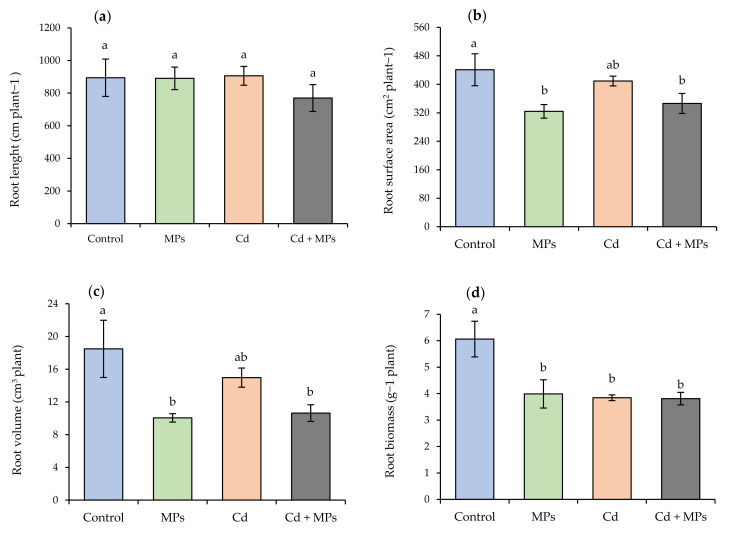
The root characteristics of strawberry plants in response to soil contamination with MPs and Cd. (**a**) Root length; (**b**) root surface area; (**c**) root volume; (**d**) root biomass. Control: no contaminants added; MPs: microplastics added; Cd: cadmium added; and Cd + MPs: a combined application of cadmium and microplastics. Different lowercase letters indicate significant differences according to the LSD Fisher test (*p* < 0.05).

Morphological parameters of the roots presented significant differences (*p* < 0.05) in surface area and volume (Figure 3). The control treatment was statistically different from the MPs and Cd + MPs treatments, with considerably higher average values of 440.8 cm^2^ in root surface area and 18.4 cm^3^ in root volume. On the other hand, no significant differences were found in root length between the treatments. However, the Cd + MPs treatment had lower average values than the other treatments, with decreases of 13.87, 13.48, and 15.01% compared to the control, Cd, and Cd + MPs treatments, respectively. In terms of root biomass, significant differences (*p* < 0.05) were observed between the control and the other treatments, with reductions of 35.0, 36.6, and 36.5% for the MPs, Cd, and Cd + MPs treatments, respectively (Figure 3).

### 2.5. Soil Biological Properties

Significant differences (*p* < 0.05) were found in the activity of acid phosphatase among the treatments (Table 2). The Cd + MPs treatment reached 39.9 μmol PNP g^−1^h^−1^, being statistically different from the control and Cd treatments, but not differing with respect to the MPs treatment. In terms of urease activity, there were no significant differences among the treatments, recording similar average values. On the other hand, the treatments with separate and combined applications of Cd (Cd and Cd + MPs) had lower dehydrogenase activity, being statistically different from the control (39.5 μg INTF g^−1^), and MPs treatments (37.1 μg INTF g^−1^). In terms of microbial biomass, significant differences were observed between the treatments containing MPs (MPs and Cd + MPs) with respect to the control and Cd treatments. Conversely, no significant differences were observed between the treatments in terms of soil respiration. However, higher values were observed in the control and MPs treatments.

### 2.6. Cd Concentration in the Roots and Soil

**Figure 4:**
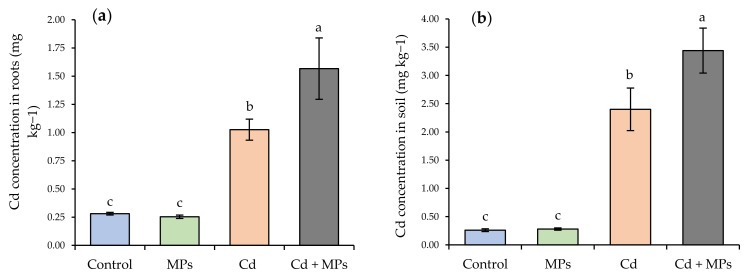
(**a**) Cadmium concentration (mg kg^−1^ dry weight) in strawberry roots for each treatment. (**b**) The concentration of cadmium in the soil for each treatment. Control: no contaminants added; MPs: microplastics added; Cd: cadmium added; and Cd + MPs: a combined application of cadmium and microplastics. Different lowercase letters indicate significant differences according to the LSD Fisher test (*p* < 0.05).

At the root level (Figure 4a), there were significant statistical differences between the treatments. The Cd + MPs treatment recorded the highest Cd concentration of 1.56 mg kg^−1^, while values for rest of the treatments were 34.62% (Cd), 82.05% (control), and 83.97% (MPs) lower. In addition, the Cd treatment was statistically different from the control and MPs treatments, with concentrations that were 77.78% and 80.16% lower, respectively. Furthermore, a similar pattern was observed in the concentration of Cd in the soil (Figure 3b). The Cd + MPs treatment recorded the highest Cd concentration of 3.44 mg kg^−1^, while values for rest of the treatments were 29.41% (Cd), 91.79% (control), and 92.35% (MPs) lower. Furthermore, the Cd treatment was statistically different from the control and MPs treatments, with concentrations that were 87.17% and 88.33% lower, respectively.

### 2.7. Correlations

**Figure 5:**
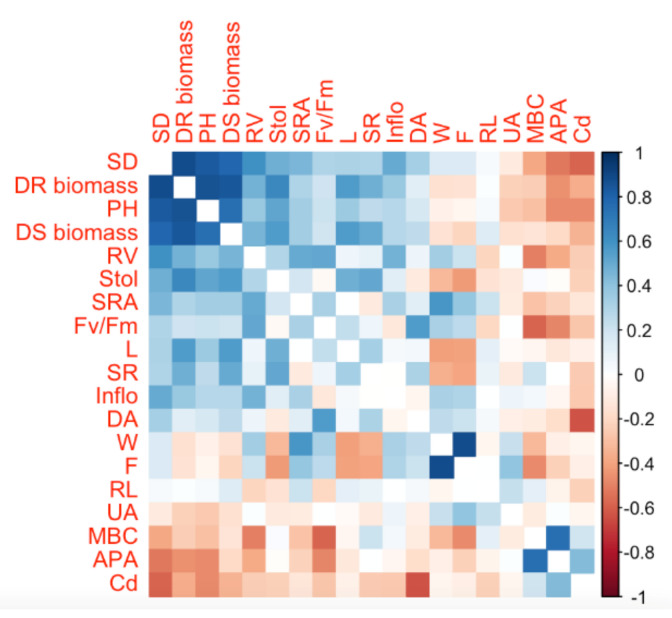
The correlation matrix for soil variables and plant performance parameters. SR: soil respiration; MBC: microbial biomass C; DA: dehydrogenase activity; UA: urease activity; APA: acid phosphatase activity; Cd: cadmium concentration in the soil; Inflo: the number of inflorescences; Stol: the total number of stolons; L: stolon length; F: the total number of fruit; W: fruit weight; DR biomass: dry root biomass; DS biomass: dry stem biomass; RL: root length; SRA: root surface area; RV: root volume; Fv/Fm: the maximum quantum yield of PSII II; PH: plant height; and SD: stem diameter.

A correlation matrix was performed to study the effects on plant soil properties and plant physiological and morphological parameters in response to the addition of contaminants (Figure 5). Cd content was negatively correlated with biomass; the higher the level of biomass, the lower the Cd content. Furthermore, Cd content was negatively correlated with the rest of the parameters, but positively correlated with acid phosphatase activity (APA) and microbial biomass C (MBC). Soil properties such as microbial biomass C (MBC) presented a positive correlation with soil respiration (SR), the number of inflorescences (Inflo), root length (RL), acid phosphatase activity (APA), and Cd content. The matrix of correlations shows that plant performance parameters such as the total number of stolons (Stol), stolon length (L), total number of fruit (F), and fruit weight (W) are highly correlated.

**Figure 6:**
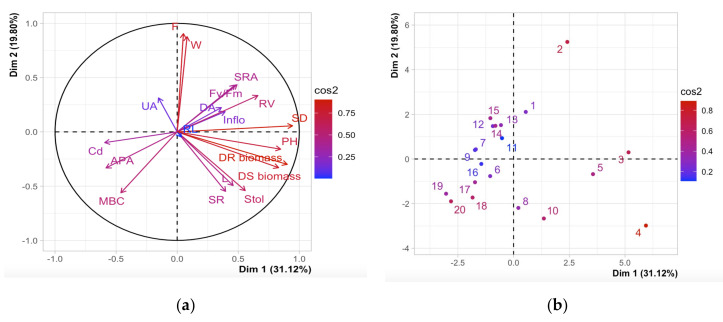
Principal component analysis (PCA) of SR: soil respiration; MBC: microbial biomass C; DA: dehydrogenase activity; UA: urease activity; APA: acid phosphatase activity; Cd: Cd concentration in the soil; Inflo: the number of inflorescences; Stol: the total number of stolons; L: stolon length; F: the total number of fruit; W: fruit weight; DR biomass: dry root biomass; DS biomass: dry stem biomass; RL: root length; SRA: root surface area; RV: root volume; Fv/Fm: the maximum quantum yield of PSII II; PH: plant height; and SD: stem diameter. (**a**) PCA of variables. (**b**) PCA of individuals.

PCA analysis (Figure 6) was performed for 19 parameters: soil respiration, microbial biomass, dehydrogenase activity, urease activity, acid phosphatase activity, cadmium concentration in the soil, the number of inflorescences, the total number of stolons, stolon length, the total number of fruits, fruit weight, root dry biomass, stem dry biomass, root length, root surface area, root volume, Fv/Fm, plant height, and stem diameter. PC1 and PC2 retained 31.12% and 19.80%, respectively. This represents all parameters as vectors in the biplot below (Figure 3a), while vector length shows how well-represented the variables are in this graph. These results confirm what was observed in the correlation matrix. Treatments for strawberry plants in the PCA of individuals are represented by the numbers 1–5 for the untreated control, 6–10 for the MPs treatment, 11–15 for the Cd treatment, and 16–20 for the Cd + MPs treatment.

## 3. Discussion

### 3.1. Plant Growth and Yield

The addition of MPs significantly decreased plant height and stem diameter. These results confirm and expand the findings of previous works on wheat [8], broad beans [26], and common beans [27], which describe the negative effects of MPs on plant and root growth. Regarding stem and root dry biomass, there were significant differences (*p* < 0.05) between the treatments containing soil pollutants (MPs, Cd, and Cd + MPs). However, no differences were found in terms of the number of inflorescences, the number of stolons, stolon length, and root length. It has been documented that the presence of MPs does not affect plant parameters such as plant biomass [28], but this is the first study evaluating the effect of MPs on the number of inflorescences, fruits, and stolons.

The results of the present study have shown that root growth (volume, area, and biomass) decreases with MPs. This agrees with the literature, which has shown an effect on the development and growth of roots [29]. However, other studies [23] have reported increases in root biomass in the presence of Cd and decreases in the presence of polyethylene MPs. In our study, plant biomass decreased in the treatments with MPs, Cd, and Cd + MPs added. In addition, root development showed a decrease in root surface area and root volume, which can be explained by the plant’s response (root and stem) to the concentration of Cd. Upon completion of the study, a higher concentration of Cd was found in the roots and soil. This suggests that MPs amplify the bioavailability of soil Cd and Cd bioaccumulation. It has been previously reported that Cd pigments from MPs are easily released in hydroponic systems (such as the ones in this study) through regulated photodissolution processes mediated by sunlight [30]. Therefore, this could explain the increase of almost 45% in Cd availability in the soil with MPs added, compared to the rest of the treatments (Figure 4b). It should be noted that the contribution of Cd to the soil, or the kinetics of Cd release, depends largely on the size of the MPs [31]. In addition, the presence of MPs in the soil–plant system results in a decreasing adsorption capacity of metals and an increasing desorption capacity of metals, which means higher bioavailability of metals to soil biota [31]. The retention capacity of heavy metals depends on the type and size of plastic particles [32], evidencing that strong hydrophobicity and high specific surface area are responsible for the accumulation of heavy metals in MPs [31]. It has been shown that the accumulation of heavy metals in plants is highly related to the concentrations of bioavailable heavy metals [33]. In the present study, plant biomass was related to plant height, showing significant differences between the treatments with contaminants and the control. This suggests that intoxication with Cd [34], whose bioavailability increased due to the presence of MPs, caused stress to the plant. MPs can also increase the bioavailability of Cd in the soil by altering soil properties and occupying sorption sites, which keeps labile MPs mobilized and prevents stabilization [13], thus promoting the mobilization, release, and subsequent accumulation of Cd in the plant [35]. Another factor that may influence the availability of Cd is soil organic matter. In this sense, it has been reported that organic matter reduces the bioavailability of Cd through the formation of stable complexes with abundant functional groups and Cd [36,37]. In our study, the level of organic matter was low (3.41%), which could have increased the availability of Cd [36]. In addition, the pH was 5.61, which suggests that the effect of this parameter and organic matter affected the bioavailability of Cd and the development of the plant by ion exchange, co-precipitation adsorption, and complexation [37].

Cd strongly inhibits the synthesis of chlorophylls and their stable binding to proteins, directly affecting the photosynthetic apparatus, particularly photosystem II (FII) [38]. In our study, FII yield (Fv/Fm) was slightly lower in the Cd + MPs treatment with respect to the other treatments. This can be explained by the stress produced by an increased bioavailability of Cd due to the presence of MPs. However, some plants may develop adaptive and protective mechanisms against Cd stress, such as combining Cd with suberin, pectin, and other compounds in the cell wall location, or being sequestered in vacuoles, which may restrict Cd mobility and reduce its toxicity [39]. Furthermore, some hormones in the plant, such as auxin, ethylene, or brassinosteroids, can form signal transduction cascades, which may contribute to an increase in the plant’s Cd tolerance [40]. In this sense, the plant response observed in this study indicates that strawberry plants were able to adapt to Cd stress since only the Cd + MPs treatment showed a decrease in photosystem II on days 10, 20, 100, and 110.

### 3.2. Soil Parameters

Heavy metals inhibit enzyme activities mainly by competing for the enzyme active sites within the substrate, denaturing the enzyme protein, and/or forming a covalent bond with enzyme–substrate complexes [41]. In our study, soil enzyme activity varied depending on the type of contaminant added (Table 2). The addition of Cd caused a greater reduction in dehydrogenase activity than that observed with the separate addition of MPs. This is explained by the fact that dehydrogenase activity is one of the most important and sensitive bioindicators of soil contamination by heavy metals [42]. One of the reasons why it was highly and negatively affected by Cd, as observed in the Cd and Cd + MPs treatments, is that Cd can alter enzyme activity by binding to its functional groups (sulfhydryl, carboxyl, imidazole, etc.) and/or by displacing other metal ions associated with it, leading to complex enzyme mechanisms in response to Cd stress [43]. In addition, the kinetic properties of dehydrogenase can also be affected by the presence of Cd, limiting the catalytic reaction of this enzyme [44].

With respect to urease activity, no significant differences were found between the treatments (Table 2) since this parameter was not sensitive to the contamination and/or stress by Cd or HDPE MPs. This contrasts with what is found in other studies [45], which report significant differences in urease activity under polyethylene MPs contamination. The activity of this enzyme is closely related to the nitrogen cycle in soil, which particularly promotes the hydrolyzation of nitrogen-containing organic matter [46]. In our study, the high levels of available N (82.2 mg kg^−1^ in the form of NH^4+^ or NO^−3^) found in the substrate can account for the decrease in urease activity [47]. Regarding acid phosphatase activity, higher activity was found in the Cd + MPs treatment, which can be explained by the reduction in the amount of available P derived from the higher bioavailability of Cd [25]. However, this differs from other studies, which have reported that soil enzyme activities decrease with an increasing availability of heavy metals [48], indicating that Cd has an inhibitory effect on phosphatase [49]. With respect to microbial biomass, the factor that most affected this parameter was the presence of MPs (MPs; Cd + MPs). In fact, MPs can be another carbon source for soil microorganisms, and serve as new substrates for the colonization of microorganisms [50]. The Cd treatment negatively affected soil microbial biomass, but no significant differences were observed with respect to the control. This may be associated with the fact that microbial adaptation processes require more energy in the presence of Cd [51], decreasing the conditions required for the permanence and development of microorganisms in the substrate [52]. In the control treatment, the plants might have consumed the available nutrients in the soil which are necessary for the development of microbial activity in the soil [53]. This is related to plant height and stem diameter since greater vegetative growth and larger amounts of stem and root biomass were observed in the control treatment with respect to the other treatments. Soil respiration has been described as a good indicator of total soil microbial activity [54]. However, although lower values were obtained in the Cd and Cd + MPs treatments, there were no significant differences between the treatments. Soil respiration can be inhibited in the presence of heavy metals [55], probably because the toxicity generated by heavy metals can suppress or even kill sensitive soil microorganisms, and thus cause a decrease in soil respiration [55]. In our study, the dose of the contaminant is responsible for the fact that rates tended to be low, but no significant differences were observed between the treatments.

### 3.3. Influence of Variables

In the principal component analysis (Figure 6a), a negative correlation was observed between the concentration of Cd in the soil and the maximum photochemical efficiency of PSII, which agrees with previous studies [56]. Negative correlations were also observed between Cd and root volume, root surface area, the number of inflorescences and dehydrogenase activity, and the high extraction of Cd from the soil by the plant, affecting physiological functions and the homeostasis of a great variety of horticultural crops [57]. The negative correlation between the concentration of Cd in the soil and dehydrogenase activity confirms the results found in our study and agrees with another study that has reported that the activity of this enzyme decreases in the presence of Cd [58]. A positive correlation was found between Cd in the soil and microbial biomass (Figure 6a), reaffirming the results found in our study (Table 2). This agrees with other works that have shown that the presence of Cd has inhibitory or lethal effects on microorganisms [59], affecting the decomposition of organic matter, the cycle of nutrients, and the use of plant nutrients [60]. This may be related to the variations in enzyme activity since MPs, Cd, or their combined application resulted in substantial effects on the biological properties of the soil.

## 4. Materials and Methods

### 4.1. Experimental Setup

A pot experiment was conducted between August 2020 and February 2021 under greenhouse conditions, using strawberry plants (*Fragaria* x *ananassa* Duch), cv. Albion (day-neutral), and a growth substrate (soil-sand; 1:1 = Vol:Vol). The plants were obtained from the Llahuen Nursery (LLahuen Farm, Huelquen Paine, Metropolitan Region, Chile), and were individually transplanted to 2 L-clay pots that were 20 cm high and 7 cm in radius. Earthenware plates 25 cm in diameter were placed under the pots to collect possible leachate from irrigation.

Doses of streptomycin and oxytetracycline (Strepto Plus, Enco Laboratory, Mendoza, Argentina) were applied to the plants (1.2 g L^−1^) to prevent plant pathogens.

The experiment consisted of a completely randomized design with four treatments and five replicates, for a total of twenty experimental units, where the factor evaluated was the addition of MPs, Cd, and Cd + MPs combined. The doses used were 0.2 g MPs kg^−1^ soil and 3 mg Cd kg^−1^ soil. An untreated control (no addition of MPs or Cd) was also established.

Soil moisture was maintained at 70% of water retention capacity and kept constant during the experiment by manually irrigating all of the pots.

Five months after transplanting (20 February 2021) the plants were harvested. Shoots, fruits, and roots were separated, and soil samples were collected for microbiological analysis.

### 4.2. Soil Description and Preparation

Soil samples were taken from the Experimental Station of the Universidad Adventista de Chile, Diguillin Province, Ñuble Region, Chile (Lat. 36°31′ S; Long. 71°54′ W). The soil corresponds to a Typic Melanoxerand, a volcanic ash-derived soil (Andisol) [61]. All the soil samples were mixed with autoclaved sand (50:50 *w*/*w*), air-dried, and sieved through a 2 mm sieve for the analysis of soil properties. The growth substrate (soil-sand) presented the following chemical characteristics: 82.2 mg kg^−1^ of available nitrogen, 47.6 mg kg^−1^ of available Olsen P, 220.6 mg kg^−1^ of available potassium, 17 cmol_c_ kg of exchangeable Ca, 3 cmol_c_ kg^−1^ of exchangeable Mg, 3.41% organic matter, 0.5 mg kg^−1^ of total Cd, and pH 5.61.

### 4.3. Microplastics Particles and Heavy Metals

High-density polyethylene (HDPE) was selected for the pot experiment because it is the most commonly used plastic in agricultural mulch films. This plastic was black in color, 20 µm thick, carbon black + uv additive, had a tear resistance of 6000 gm mm^−1^, and an impact resistance f50 of 200 g (SolPlast S.A., Lorca, Murcia, Spain). The MPs particles were obtained by manually cutting and sieving HDPE, obtaining particles of 2 to 5 mm.

Cadmium chloride (CdCl_2_) was used as the contaminant material, and corresponded to 99.9% elemental Cd (Sigma Aldrich Co., St. Louis, MO, USA). An amount of 3 mg L^−1^ of CdCl_2_ was applied to the soil at the beginning of the assay.

### 4.4. Plant Analysis

Maximum chlorophyll fluorescence (Fm) and minimum chlorophyll fluorescence (F0) were determined. F0 was measured on a clear day at intervals of 10 days from 15 September 2020 to 15 February 2021, and during the day at 9:00, 11:00, 13:00, 3:00, and 5:00 using a portable fluorimeter model OS-5p (Opti-Sciences, Hudson, NH, USA). Both F0 and Fm were determined after a period of 30 min in which the leaves adapted to the darkness [62]. To obtain the level of darkness, foliar clips including a movable obturation plate were used. Based on these parameters, the maximum photochemical efficiency of photosystem II (Fv/Fm) was quantified, using the ratio Fv/Fm = (Fm − F0) / Fm [63].

Plant height was measured from budding to flowering, using a millimeter ruler. The number of inflorescences was counted from 30 October 2020 to 30 January 2021, while fruits were harvested from 23 November 2020 to 20 February 2021. At harvest, the strawberries were weighed using a precision scale (Precisa instruments AG, Dietikon, Switzerland), and frozen at −20 °C.

After harvest, the strawberry plants were counted while stolon length was measured with a millimeter ruler. Plant biomass (aerial and root parts) was evaluated dry at 70 °C for 48 h and weighed. The determinations of root volume, root length, and root diameter were performed with WinRHIZO Reg software (V5.0, Regent Instrument Inc., Quebec, QC, Canada). The contents of Cd in the roots and soil were evaluated using the Espect ICP-MS technique, which uses a heat block digestion system (DigiPREP, Clark Graham Baie D’Urfé, QC, Canada).

### 4.5. Microbiological Soil Analysis

Dehydrogenase activity was determined using 1 g of soil at 60% field capacity exposed to 0.2 mL of 0.4% INT (2-p-iodoophenyl 3-p-nitrophenyl-5 phenyltetrazolium chloride) in distilled water at 22 °C in darkness for 20 h. The iodonitrotetrazolium formazan (INTF) formed was extracted with 10 mL of methanol by shaking vigorously for 1 min and filtering through Whatman N° 5 filter paper. INTF was measured spectrophotometrically at 490 nm. Dehydrogenase activity was expressed in terms of micrograms INTF per gram of soil with reference to a standard INTF curve [64].

Urease activity was determined in 0.1 M phosphate buffer at pH 7. An amount of 1 M urea was used as substrate. Aliquots of 2 mL of buffer and 0.5 mL of substrate were added to 0.5 g of the sample and then incubated at 30 °C for 90 min. Urease activity was determined as the NH^4+^ released in the hydrolysis reaction [65], based on the ammonium chloride standard curve.

Acid phosphatase activity was determined using p-nitrophenyl phosphate disodium (PNPP 0.115 M) as substrate. A volume of 2 mL of 0.5 M sodium acetate buffer at pH 6 using acetic acid [66], and 0.5 mL of substrate were added to 0.5 g of soil sieved to <2 mm and then incubated at 37 °C for 90 min. The reaction was stopped by cooling at 0 °C for 10 min. Then, 0.5 mL of 0.5 M CaCl_2_ and 2 mL of 0.5 MNaOH were added, and the mixture was centrifuged at 4000 rev. min^−1^ for 5 min. The p-nitrophenol (PNP) formed was determined by spectrophotometry at 398 nm [67].

The microbial C biomass was determined by FDA (fluorescein diacetate) hydrolysis. An amount of 1.0 g of wet soil was used; samples were prepared in triplicate and a blank was also included. Volumes of 9.9 mL of sodium phosphate buffer (60 mM; pH 7.8) and 0.1 mL of 2 mg fluorescein diacetate (FDA) mL^−1^ acetone were added to the soil samples, while a volume of 10 mL of buffer was added to the blank. The tubes were shaken in a vortex and then incubated at 20 °C for 1 h in a thermostatic bath. After incubation, samples were cooled in an ice water bath and a volume of 10 mL of acetone was added to all of the tubes (samples and blanks), which were shaken and filtered through Whatman No. 40 filter paper. After that, the absorbance of the samples and blanks was read in the spectrophotometer (Rayleigh—Model UV1601 UV/VIS, Beijing, China) at 490 nm. The results were expressed as μg fluorescein g^−1^ dry soil [68].

Soil respiration was determined using an amount of 25 g of soil (in duplicate) per treatment placed in an incubation bottle. A volume of 7.5 mL of NaOH (0.5 M) was placed in a centrifuge tube and then placed in an incubation bottle. Jars without soil (blank) were hermetically closed and remained in an incubation chamber at 22 °C and constant humidity for 7 days. After the incubation time, a volume of 1 mL of NaOH (0.5 M) was taken from the centrifuge tube and mixed with a volume of 2 mL of BaCl_2_ (1 M); phenolphthalein indicator was previously added (2 to 3 drops) to the solution. Subsequently, the solution was titrated with HCl (0.1 M) and the data were expressed as μg C-CO_2_ g^−1^ soil [68].

### 4.6. Statistical Analysis

Differences between the treatments were compared using Fisher’s LSD test at *p* < 0.05. A one-way ANOVA analysis was performed to analyze the interaction between microplastics and cadmium. All figures and graphs were generated using R and Infostat softwares [69]. Principal component analysis (PCA) was applied using mean-centered data based on the eigen values to determine the correlation between variables and characteristics in different conditions of MPs and heavy metals using R software by FactoMineR and the ggplot2 package.

## 5. Conclusions

The results of this experiment demonstrated that MPs and Cd + MPs can affect plant height, stem diameter, plant biomass, root volume, root surface area, and root biomass, as well as soil enzyme activities (acid phosphatase and dehydrogenase) and microbial biomass. High levels of Cd (Cd> 3 mg kg^-1^) combined with the presence of microplastics in the soil favor a greater accumulation of heavy metals in the cultivation of strawberry plants, decreasing the total number of fruits and total biomass per plant. According to the results obtained in this study, it is proposed to continue studying the dynamics and interaction of Cd and MPs on the microbiological diversity of soil, in addition to the translocation and bioaccumulation of Cd in the edible part of the strawberry.

## Figures and Tables

**Table 1:** Morphological and production parameters of strawberry plants in response to soil contamination with MPs and Cd. Control: no contaminants added; MPs: microplastics added; Cd: cadmium added; and Cd + MPs: a combined application of cadmium and microplastics.

Treatments	Number of Inflorescences (Flowers Plant^−1^)	Total Number of Stolons (Stolons Plant^−1^)	Stolon Length (cm Plant^−1^)	Total Number of Fruits (Fruits Plant^−1^)	Fruit Weight (g Plant^−1^)	Total Plant Biomass (g Plant^−1^)
Control	5.4 ± 0.67 a	1.6 ± 0.67 a	40.1 ± 10.10 a	4.4 ± 1.50 a	20.9 ± 8.80 a	7.3 ± 1.00 a
MPs	3.2 ± 0.80 a	1.4 ± 0.51 a	26.6 ± 8.60 a	2.0 ± 0.50 b	8.8 ± 2.80 a	4.4 ± 0.30 b
Cd	3.8 ± 0.80 a	1.0 ± 0.31 a	23.7 ± 6.70 a	3.8 ± 0.50 a	20.4 ± 2.10 a	4.2 ± 0.30 b
Cd + MPs	3.2 ± 0.37 a	1.2 ± 0.20 a	29.9 ± 3.40 a	2.0 ± 0.30 b	9.8 ± 1.20 a	4.8 ± 0.30 b
Anova *p* values	0.1165	0.9160	0.4767	0.0426	0.1783	0.0073

Different lowercase letters indicate significant differences according to the LSD Fisher test (*p* < 0.05). Mean ± standard error (n = 5).

**Table 2:** Soil microbiological properties and enzyme activity in response to soil contamination with MPs and Cd. Control: no contaminants added; MPs: microplastics added; Cd: cadmium added; and Cd + MPs: a combined application of cadmium and microplastics.

Treatments	Acid Phosphatase Activity (μmol PNP g^−1^h^−1^)	Urease Activity (μmol NH_4_^+^g ^−1^h^−1^)	Dehydrogenase Activity (μg INTF g^−1^)	Microbial Biomass C (µg Kg^−1^)	Soil Respiration (μg CO_2_ g^−1^ Day)
Control	21.3 ± 1.8 b	2.2 ± 0.10 a	39.5 ± 2.70 a	1.5 ± 0.2 b	81.5 ± 11.2 a
MPs	29.3 ± 2.7 ab	2.1 ± 0.09 a	37.1 ± 5.20 a	2.5 ± 0.2 a	85.8 ± 8.3 a
Cd	25.6 ± 2.4 b	2.3 ± 0.10 a	23.2 ± 2.08 b	1.2 ± 0.2 b	69.9 ± 3.4 a
Cd + MPs	39.9 ± 3.8 a	2.1 ± 0.10 a	22.9 ± 4.80 b	3.1 ± 0.4 a	72.3 ± 3.6 a
Anova *p* values	0.001	0.5948	0.0125	0.0023	0.4138

Different lowercase letters indicate significant differences according to the LSD Fisher test (*p* < 0.05). Mean ± standard error (n = 5).

## Data Availability

The data presented in this study are available in article.
